# Immune-Related lncRNA Pairs as Prognostic Signature and Immune-Landscape Predictor in Lung Adenocarcinoma

**DOI:** 10.3389/fonc.2021.673567

**Published:** 2022-01-10

**Authors:** Zhengrong Yin, Mei Zhou, Tingting Liao, Juanjuan Xu, Jinshuo Fan, Jingjing Deng, Yang Jin

**Affiliations:** Department of Respiratory and Critical Care Medicine, NHC Key Laboratory of Pulmonary Diseases, Union Hospital, Tongji Medical College, Huazhong University of Science and Technology, Wuhan, China

**Keywords:** immune-related lncRNA pair, lung adenocarcinoma, signature, immune landscape, drug sensitivity

## Abstract

**Background:**

Suppressive tumor microenvironment is closely related to the progression and poor prognosis of lung adenocarcinoma (LUAD). Novel individual and universal immune-related biomarkers to predict the prognosis and immune landscape of LUAD patients are urgently needed. Two-gene pairing patterns could integrate and utilize various gene expression data.

**Methods:**

The RNA-seq and relevant clinicopathological data of the LUAD project from the TCGA and well-known immune-related genes list from the ImmPort database were obtained. Co-expression analysis followed by an analysis of variance was performed to identify differentially expressed immune-related lncRNA (irlncRNA) (DEirlncRNA) between tumor and normal tissues. Two arbitrary DEirlncRNAs (DEirlncRNAs pair) in a tumor sample underwent pairwise comparison to generate a score (0 or 1). Next, Univariate analysis, Lasso regression and Multivariate analysis were used to screen survival-related DEirlncRNAs pairs and construct a prognostic model. The Acak information standard (AIC) values of the receiver operating characteristic (ROC) curve for 3 years are calculated to determine the cut-off point for high- or low-risk score. Finally, we evaluated the relationship between the risk score and overall survival, clinicopathological features, immune landscape, and chemotherapy efficacy.

**Results:**

Data of 54 normal and 497 tumor samples of LUAD were enrolled. After a strict screening process, 15 survival-independent-related DEirlncRNA pairs were integrated to construct a prognostic model. The AUC value of the 3-year ROC curve was 0.828. Kaplan–Meier analysis showed that patients with low risk lived longer than patients with high risk (p <0.001). Univariate and Multivariate Cox analysis suggested that the risk score was an independent factor of survival. The risk score was negatively associated with most tumor-infiltrating immune cells, immune score, and microenvironment scores. The low-risk group was correlated with increased expression of ICOS. The high-risk group had a connection with lower half inhibitory centration (IC50) of most chemotherapy drugs (e.g., etoposide, paclitaxel, vinorelbine, gemcitabine, and docetaxel) and targeted medicine—erlotinib, but with higher IC50 of methotrexate.

**Conclusion:**

The established irlncRNA pairs-based model is a promising prognostic signature for LUAD patients. Furthermore, the prognostic signature has great potential in the evaluation of tumor immune landscape and guiding individualized treatment regimens.

## Introduction

Globally, lung cancer remains the main cause of cancer death (1). Lung adenocarcinoma (LUAD), as the most common pathological type of lung cancer, has brought great burden to the health care systems (2). The prognosis for LUAD is generally poor in virtue of the characteristics of early metastasis. Chemotherapy and molecular targeted therapy are already conventional treatments for LUAD (3). Suppressive tumor microenvironment is closely related to the progression and poor prognosis of lung cancer (4). Immunotherapy targeted to relevant immunological mechanisms especially immune checkpoint inhibitor treatment has brought promising future for cure of LUAD patients (3, 5, 6). However, individual immune heterogeneity, namely, various immune cell compositions and immunoregulatory molecules, are related to different responses to immunotherapy (6, 7), chemotherapy (8), and targeted therapy (9). It is necessary and theoretically feasible to find immune-related biomarkers that can predict the prognosis and treatment sensitivity of LUAD patients.

Long noncoding RNAs (lncRNAs, ncRNAs that are >200 nt in length), a crucial class of pervasive genes playing a variety of cellular and physiologic functions, are known to be related to tumorigenesis and metastasis (10). LncRNAs, especially immune-related lncRNAs, have been indicated to possess great potential as novel biomarkers for the prognosis and treatment effect of lung cancer (11–15). However, the prognostic signatures in these studies were found based on the exact expression level of immune-related lncRNAs. Of these methods, the process of the normalization of lncRNAs expression from different platforms made data processing complicated and might affect the accuracy of the prediction model.

An inspiring research developed and validated an individualized immune prognostic signature for lung cancer using a strategy of immune-related gene (irgene) pairing in each sample. This gene-pairing strategy left out the normalization of data from diverse platforms (16).

In the present study, we retrieved irlncRNA data of LUAD patients from the Cancer Genome Atlas (TCGA) to establish and verify an individualized and multiple-data applicable prediction model for LUAD by applying the irlncRNA-pairing strategy. Furthermore, we investigated the value of the prognostic model in evaluating the immune landscape and prediction of effects of chemotherapy and targeted therapy.

## Methods

### Data Processing and Extraction of Differentially Expressed Immune-Related lncRNAs

The RNA-seq and matched clinicopathologic data of LUAD were downloaded from the TCGA database. The mRNAs and lncRNAs were distinguished by annotation files from the Ensembl databank for subsequent analysis. The list of confirmed immune-related genes (irgenes) was downloaded from the ImmPort databank. Immune-related lncRNA (IrlncRNAs) were acquired through co-expression analysis between irgenes and lncRNAs with correlation coefficients >0.4 and the p-value <0.001 as thresholds. The “limma” R package was used to discriminate the differential expressed irlncRNAs (DEirlncRNA) between tumor and normal tissues with the thresholds set as log fold change (logFC) >1 and false discovery rate (FDR) <0.05. Patients lacking clinicopathological data and those with survival data <30 days (who may die of other diseases rather than LUAD) were excluded.

### Pairing DEirlncRNA

The DEirlncRNAs in each tumor sample were randomly paired. The score of DEirlncRNAs pair (DEirlncRNA 1/DEirlncRNA 2) was assigned to be 1 if the expression of DEirlncRNA 1 was more than DEirlncRNA 2; otherwise, the DEirlncRNAs pair score was 0 (16). Therefore, we constructed a 0-or-1 matrix. DEirlncRNAs pair score with constant values (0 or 1 over 80% frequency or under 20%) was considered not associated with prognosis because a certain rank was necessary for the survival of the discriminating patient (17). Accordingly, only if the frequency of value (0 or 1) of a DEirlncRNA pair score was between 20 and 80% of the total sample, it would be regarded as candidate for prognostic model construction.

### Construction and Validation of a Prognostic Model

For screening alternative DEirlncRNA pairs to construct a prognostic model, we first performed a Univariate Cox proportional hazard analysis to screen survival-related DEirlncRNA pairs. Then, a 10-fold cross-validation Lasso regression (18) was performed to further filter meaningful DEirlncRNA pairs (p <0.05 as significance threshold), which were then selected for Multivariate Cox proportional hazard analysis for the construction of the model with the risk-formula: Risk score = score of DEirlncRNA pairs 1 × β1 DEirlncRNA pairs 1 + score of DEirlncRNA pairs 2 × β2 DEirlncRNA pairs 2 +……+ score of DEirlncRNA pairs n × βn DEirlncRNA pairs n. We drew the ROC curves of the model for 1, 3, and 5 years and evaluated the Acak information criterion (AIC) values of each point of the 3-year ROC curve to determine the cut-off point for high- or low-risk score. Kaplan–Meier analysis showing the difference of survival between the high-risk group and low-risk group was performed to verify this critical value. The relation of risk score values to survival status was also explored. The R packages performed in the above steps included glmnet, survival, survminer, survivalROC, pbapply, and pHeatmap.

For the validation of the clinical significance of the constructed model, chi-square test was used to explore the relationship between risk score and clinical data. The band diagram was plotted for visualization (p <0.001 = ***, p <0.01 = **, and p <0.05 = *). We performed Wilcoxon signed-rank test to show the risk score differences among various groups divided by clinical characteristics, which were shown by the box diagram. To confirm whether the risk score can be used as an independent risk-stratification factor, Univariate and Multivariate Cox regression analyses were conducted between the risk score and clinical features. Forest maps were utilized to display the results. These procedures were utilized by the R packages, namely, Survival and pHeatmap.

### Evaluation of Tumor Immune Microenvironment Using Prognostic Signature

First, to investigate the association between the risk score and immune cells in the tumor microenvironment, we estimated the infiltrating-immune cells among the samples by acknowledged algorithms, namely, CIBERSORT (19), CIBERSORT-ABS (20), TIMER (21), xCELL (22, 23), MCPcounter (24), QUANTISEQ (25), and EPIC (26). Wilcoxon signed-rank test was used to inspect the differences of the infiltrating immune cells between high- and low-risk groups, of which the results were shown in the box chart. The Spearman correlation analysis between the risk score and the immune cells was performed and the correlation coefficients were shown in a lollipop diagram (p <0.05 as significance threshold). The operation was utilized by the R ggplot 2 package. Second, Wilcoxon signed-rank test was applied to study the differential expression of immune checkpoint-related gene between the high- and low-risk groups. Package “ggstatsplot” was performed and the violin plot was visualized.

### Assessment of the Value of the Signature in Predicting Drug Susceptibility

To evaluate the value of the signature in the LUAD treatment efficacy prediction, we counted the IC50 of common chemotherapy and molecular targeted drugs for each sample using pRRophetic (an R package for prediction of clinical chemotherapeutic response from tumor gene expression levels) (27). Antitumor medicines such as etoposide, paclitaxel, vinorelbine, docetaxel, methotrexate, erlotinib, gefitinib, crizotinib, and alectinib are recommended for LUAD treatment in guidelines (3). The difference in the IC50 between the high- and low-risk groups was compared by Wilcoxon signed-rank test and the results are shown as box drawings using a “ggplot2” R package.

## Results

### Screening of DEirlncRNAs

**Figure 1:**
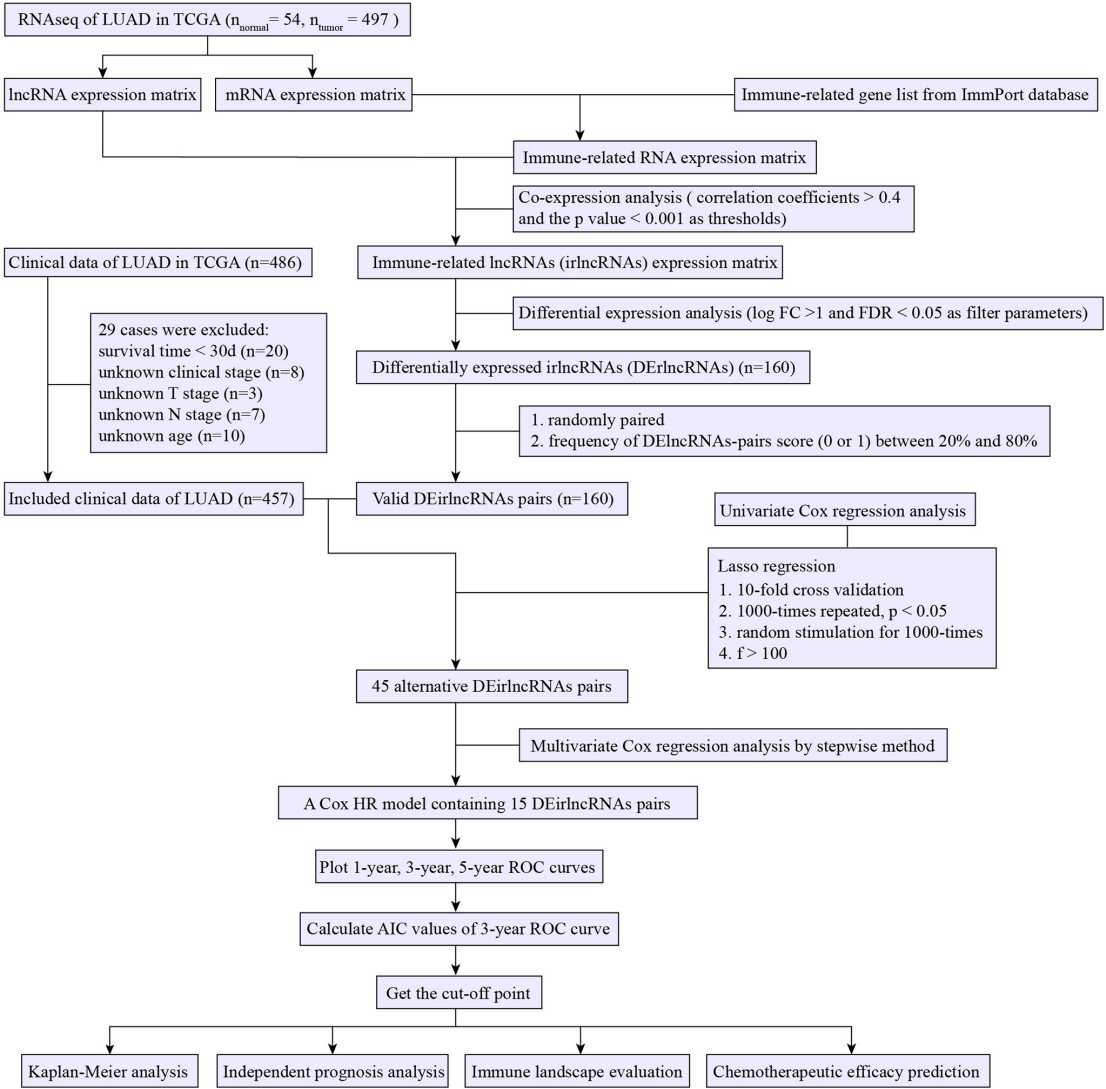
Flow chart of the study.

**Figure 2:**
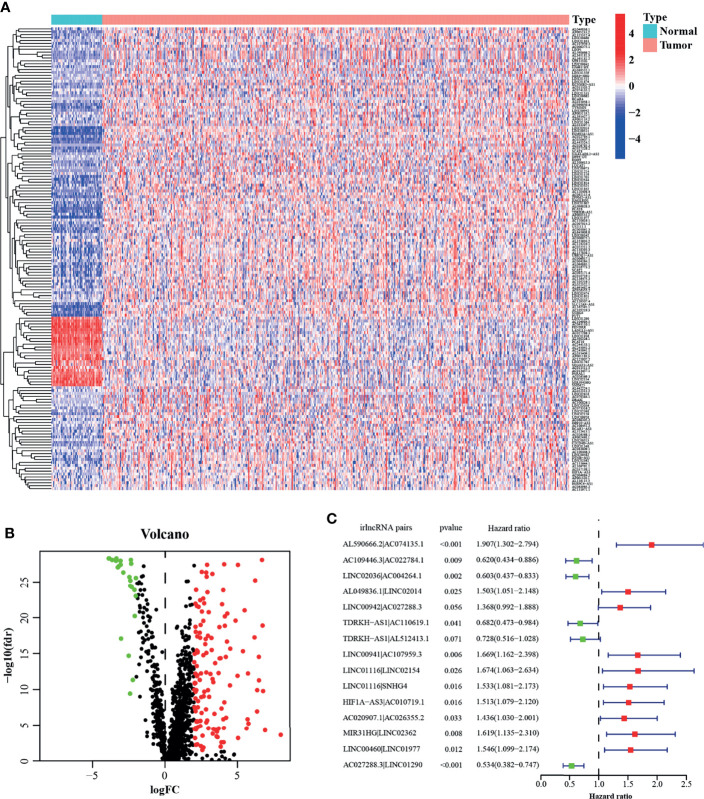
Construction of a prognostic model using DEirlncRNA Pairs. The heatmap **(A)** and volcano plot **(B)** of identified differentially expressed immune-related lncRNAs (DEirlncRNAs). **(C)** A forest map showed 12 DEirlncRNA pairs identified by Multivariate Cox proportional hazard regression in the stepwise method.

**Figure 1** presents the flow chart of this research. First, we obtained the RNA-seq data of LUAD from the TCGA database, namely, 54 normal and 497 tumor samples. Next, the data were divided into lncRNA and mRNA, and the known irgenes profiles were retrieved from the ImmPort database, then the irlncRNAs were obtained by performing co-expression analysis between irgenes and lncRNAs. Finally, we identified 1,209 irlncRNAs (shown in **Table S1**) and 160 DEirlncRNAs (**Figure 2A** and **Table S2**), among which, 136 were raised, and 24 were reduced in tumor comparing to normal tissues. (**Figure 2B**).

### Construction and Validation of Prognostic Model Based on DEirlncRNAs Pairs

**Figure 3:**
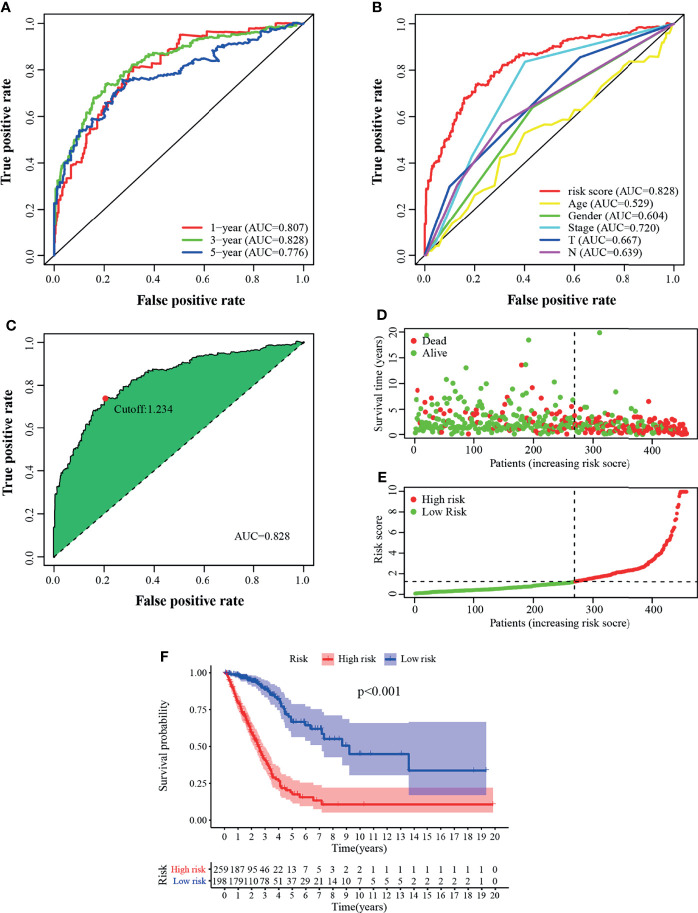
Validation of the prognostic model. **(A)** The 1-, 3-, and 5-year ROC of the model suggested that all AUC values were over 0.77. **(B)** A comparison of ROC curves of 3-year with other common clinical factors showed the superiority of the riskscore. **(C)** Riskscore for 457 patients with LUAD; the maximum inflection point is the cut-off point obtained by the AIC. Risk scores **(D)** and survival outcome **(E)** of each case are shown. **(F)** Patients in the low-risk group experienced a longer survival time tested by the Kaplan–Meier test.

A total of 457 cancer cases with survival time >30 days and matched clinicopathological features (except for M stage for 26.5% of them were missing or unknown) from the TCGA was included for the following analysis. Using an iteration loop and a 0-or-1 matrix screening among 160 DEirlncRNAs, 9,931 valid DEirlncRNA pairs were identified. After a Univariate Cox proportional hazard analysis, 260 DEirlncRNA pairs were extracted. Then a modified Lasso regression analysis was utilized to prevent overfitting and screened to 45 DEirlncRNA pairs, followed by a Multivariate Cox proportional hazards analysis, and 15 of them were incorporated into the prognostic model based on step-by-step approach. (**Figure 2C**). We drew the ROC curves of the model for 1, 3, and 5 years with all AUC values more than 0.77 and the greatest AUC value—0.828 for 3 years (**Figure 3A**). Additionally, we compared the ROC curves of the model and other clinicopathologic factors for 3 years, which showed the risk score possessed the maximum AUC value (**Figure 3B**). These results validated the optimality of the signature. We calculated the AIC value to identify the cut-off point of the ROC curve for 3 years. (**Figure 3C**). Included 457 cases were classified into 259 high-risk and 198 low-risk cases based on the above cut-off point. **Figures 3D, E** showed their risk score and survival condition. These results manifested better clinical outcome of low-risk patients than that of high-risk. A Kaplan–Meier analysis showed that patients with low risk lived longer than patients with high risk (p <0.001) (**Figure 3F**).

### Correlation Between Risk Score and the Clinical Variables

**Figure 4:**
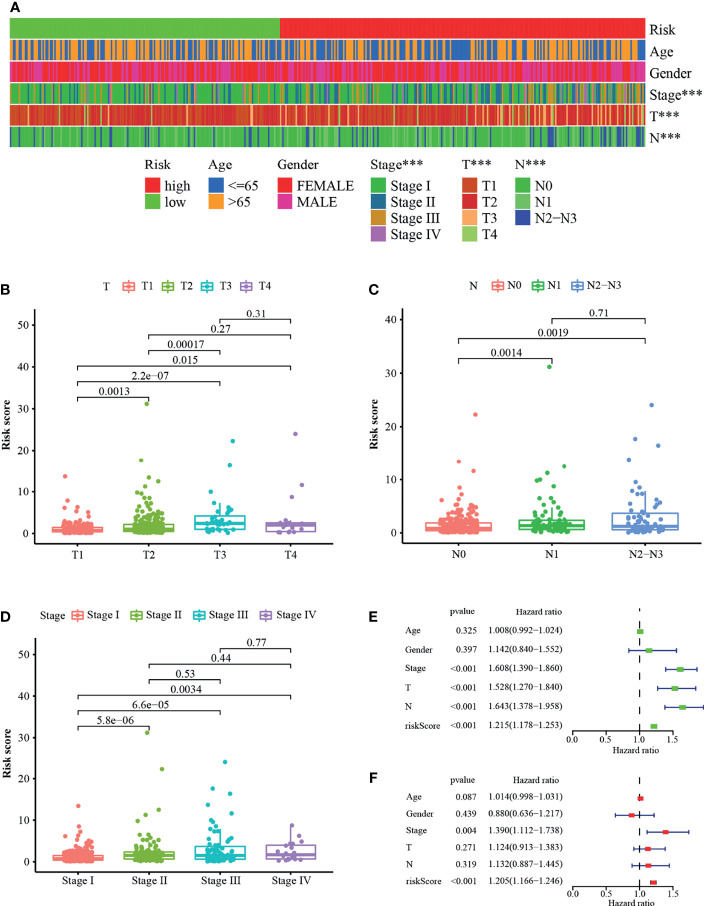
Correlation between riskscore and the clinical variables. **(A)** A strip illustration and scatter drawing showed that the **(B)** T stage, **(C)** N stage and **(D)** clinical stage were significantly related to the riskscore. **(E)** Univariate Cox hazard ratio analysis and **(F)** Multivariate Cox regression analysis of riskscore and other common clinical factors. ***p < 0.001.

The strip illustration (**Figure 4A**) and scatter drawing showed that the T stage (**Figure 4B**), N stage (**Figure 4C**), and clinical stage (**Figure 4D**) were significantly related to the risk score. Next, Univariate Cox regression analysis indicated that the clinical stage (p <0.001, HR = 1.608, 95% CI [1.390–1.860]), T stage (p <0.001, HR = 1.528, 95% CI [1.270–1.840]), N stage (p <0.001, HR = 1.643, 95% CI [1.378–1.958]), and risk score (p <0.001, HR = 1.215, 95% CI [1.178–1.253]) were associated with overall survival (**Figure 4E**), however, only clinical stage (p = 0.004, HR = 1.390, 95% CI [1.112–1.738]) and risk score (p <0.001, HR = 1.205, 95% CI [1.166–1.246]) illustrated independent correlation by Multivariate Cox regression analysis (**Figure 4F**). Overall, the risk score was an independent factor associated with survival of the patients.

### Relevance of the Prognostic Signature to Immune Landscape

**Figure 5:**
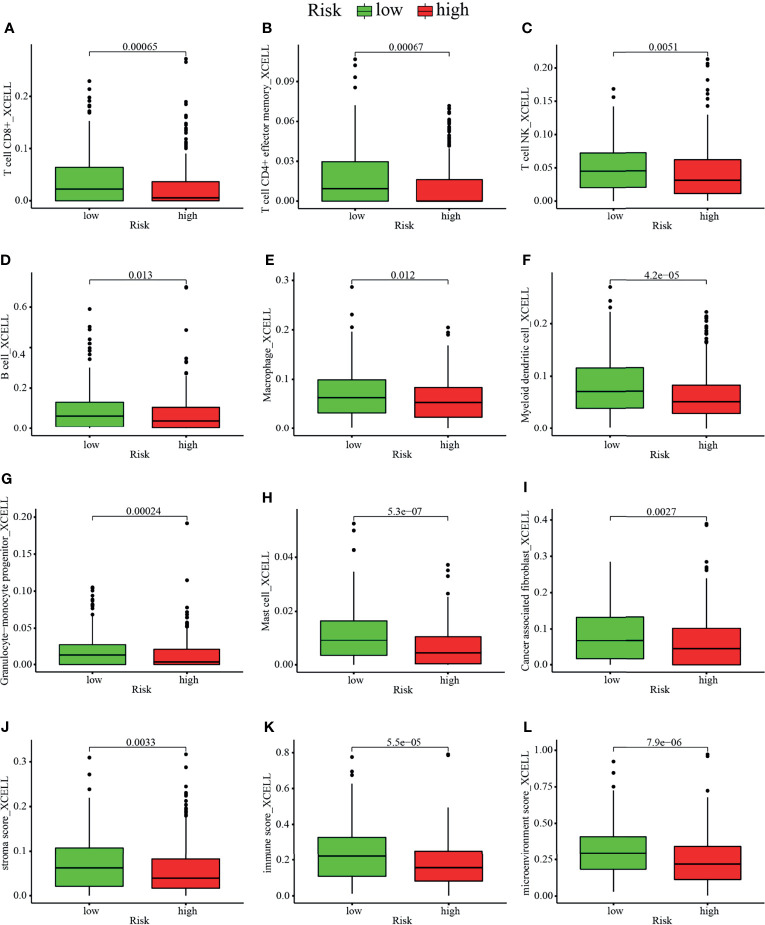
Estimation of tumor-infiltrating cells by the prognostic model. Comparison of composition of **(A–H)** immune cells, namely, **(A)** CD8^+^ T cell, **(B)** CD4^+^ effector memory T cell, **(C)** NKT cells, **(D)** B cell, **(E)** macrophage, **(F)** myeloid dendritic cell, **(G)** granulocyte–monocyte progenitor cell, and **(H)** mast cell and **(I)** cancer associated fibroblast cell between the high risk and low-risk group. **(J–L)** Comparison of **(J)** stromal score, **(K)** immune score, and **(L)** microenvironment score between the high risk and low-risk groups.

**Figure 6:**
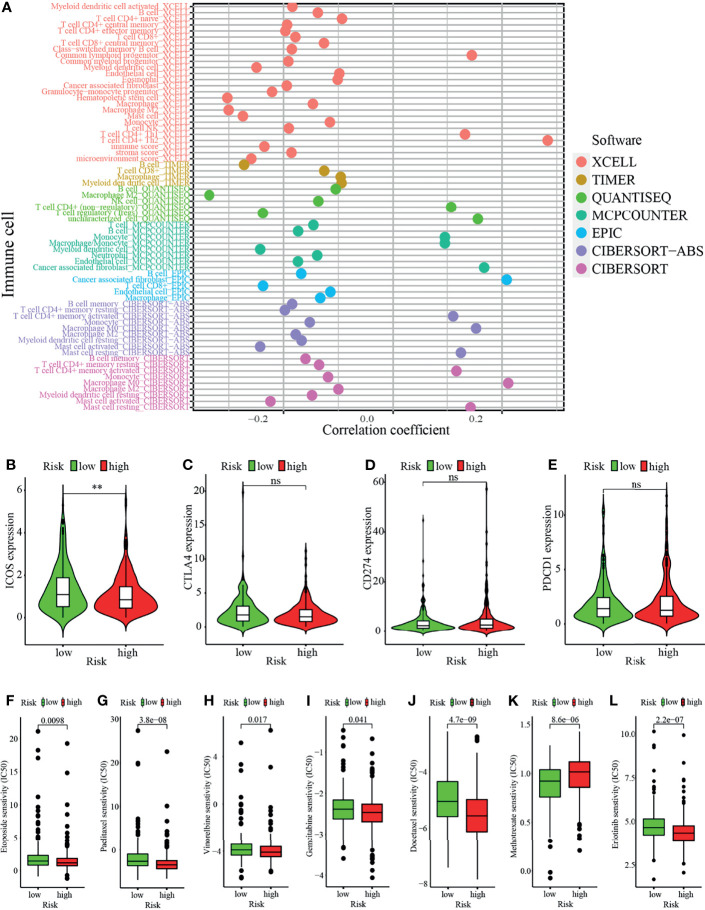
Analysis of immune landscape between the high-risk and low-risk groups. **(A)** Overview of association among riskscore and immune cells and stromal cells shown by Spearman correlation analysis. **(B–E)** Comparison of expression level of **(B)** ICOS, **(C)** CTLA4, **(D)** CD274, and **(E)** PDCD1 levels. **(F–L)** Prediction of drug sensitivity (IC50) for chemotherapeutics such as **(F)** etoposide, **(G)** paclitaxel **(H)** vinorelbine, **(I)** gemcitabine, **(J)** docetaxel, **(K)** methotrexate, and **(L)** targeted therapy—erlotinib. **p < 0.01; ns, not significant.

Studies have shown that the suppressive tumor immune microenvironment is a hallmark of tumors (including lung cancer). A breakthrough has been made in immunotherapy (cell adoptive therapy and immune checkpoint blocking therapy). We subsequently explored whether the prognostic signature based on irlncRNAs pairs had a relation to the tumor immune landscape. Results showed that most immune cells in tumor microenvironment including CD8^+^ T cells, CD4^+^ T cells, monocytes, B cells, dendritic cells, and NKT cells were negatively associated with the high-risk scores, whereas fibroblasts hold distinct results in different algorithms (**Figures 5A**–**I**, **6A** and **Tables S3, S4**). Tumor environment score, immune, and stromal score (**Figures 5J**–**L**) calculated by xCELL algorithm were higher in the low-risk group than the high-risk group. Besides, we explored whether the prognostic signature was correlated with immune checkpoint-related gene expression, and found that the high-risk group showed a higher level of ICOS (p <0.01, **Figure 6B**), although CTLA4, CD274, and PDCD1 (**Figures 6C**–**E**) showed no significant association.

### Correlation Analysis Between the Prognostic Signature and Chemotherapeutics

In addition to immune checkpoint blockades therapy, we tried to explore whether there were associations between risk score and the sensibility of LUAD patients to the common chemotherapeutics and molecular targeted therapy. Results showed that risk score was negatively related to IC50 of chemotherapy drugs such as etoposide (p = 0.0098), paclitaxel (p <0.0001), vinorelbine (p = 0.017), gemcitabine (p = 0.041), and docetaxel (p <0.0001), whereas it was positively associated with IC50 of methotrexate (p <0.0001), which suggested that the model possessed great potential in predicting chemotherapeutic sensitivity (**Figures 6F**–**K**). In addition, the risk score was suggested to be negatively associated with the IC50 of erlotinib (p <0.0001) (**Figure 6L**) though there was no significant association between other targeted drugs (such as gefitinib or afatinib) (data not shown).

## Discussion

Lung cancer is still the most afflicting cancer in the world and the 5-year survival rate of lung cancer is only 10–20% in many countries (1). Comprehensive screening with low-dose computed tomography (CT) and advances in therapeutic strategies such as targeted therapy and immunotherapy had improved the survival of lung cancer patients. However, individual heterogeneity (e.g., immune heterogeneity) of patients results in differential responses to immunotherapy (7) and chemotherapy (8) and targeted therapy (9). Discovering immune-related biomarkers that can predict the prognosis and treatment sensitivity of LUAD patients for adjusting the optimum treatment regimens in advance was urgently needed. Recent studies have shown immune-associated lncRNAs signature has a prognostic (overall survival) value (11–14) or immunotherapeutic effect (15) for LUAD patients. However, these prognostic signatures are restricted by the normalization processing of lncRNA expression data from different platforms. In this study, we took a strategy using irlncRNA pairs, inspired by the research of Li (16), to establish and validate an individual and reliable model to predict prognosis and provide references for selection of therapeutic drugs of patients with LUAD. The founding of the prognostic model in our study is the comparative ranking of irlncRNA expression in a tumor sample, which can utilize irlncRNA expression data from various sources such as microarray, RNA-Seq, or quantitative PCR.

Prognostic signatures associated with the tumor immune landscape possess great potential in recognizing new molecular biomarkers and ameliorating patient management (28). Our prognostic model based on 15 irlncRNA pairs showed excellent performance in distinguishing high and low-risk groups. Moreover, it was an independent predictive factor for the prognosis of LUAD patients. A total of 12 of 27 irlncRNAs (15 irlncRNA pairs) in the model have been identified as biomarkers or been found to take a crucial part in the pathogenesis of cancer or other diseases. AC022784.1 (12), TDRKH-AS1 (29), and LINC00941 (29) had been reported to be associated with the prognosis of LUAD. LINC00942, LINC01116, SNHG4, MIR31HG, and LINC00460 had been known to be associated with tumor development and progression and drug resistance in various cancers including lung cancer (30–39). AC107959.3 (40) and LINC02154 (41) were reported to be associated with the prognosis of hepatocellular carcinoma and laryngeal cancer respectively. LINC01977 (42) and HIF1A-AS3 (43) might be related to the pathogenesis of thyroid carcinoma multiple sclerosis respectively, whereas other 15 irlncRNAs were revealed for the first time. Whether these new irlncRNAs are novel biomarkers and play crucial roles in LUAD progress needs further research.

The composition of tumor-infiltrating immune cells and immune checkpoints have related to the responses to immune checkpoint inhibitors (44, 45). Lung cancer patients with higher PD-L1 expression possessed a better effect of pembrolizumab therapy than those with lower expression (6). In our study, the low-risk group possessed a higher composition of most immune cells, namely, CD4^+^ T cells, CD8^+^ T cells, B cells, and dendritic cells, which was consistent with previous studies (45–47). The low-risk group had a higher microenvironment score, immune and stromal score indicated that they possessed lower tumor purity and superior responses from immunotherapy (48). The low-risk group had a higher level of ICOS expression though there was no significant relation between riskscore and expression of CTLA4, CD274 or PDCD1. These results suggested that patients of low risk might have superior responses to immunotherapy such as immune checkpoint blockade and cancer vaccines. Nevertheless, the high-risk group in our study was more sensitive to chemotherapeutics such as etoposide, paclitaxel, vinorelbine, gemcitabine and docetaxel and targeted therapeutic drug-erlotinib. Therefore, the prognostic signature in our study has great potential in guiding treatment strategies for LUAD in clinical practice.

There are several limitations to our study. First, this was a retrospective study. Second, the dataset was simply downloaded from TCGA and further experimental data is needed to support these findings. Third, we had not done external validation for the constructed model to improve its applicability, which is restricted to the potential selection bias of patients. We utilized relative ranking of irlncRNA expression values within each sample to minimize errors caused by differential expression and diverse detection platforms, and the individualized prognostic signature possessed certain applicability for its ability to integrating various data sources from microarray, RNA-Seq or real-time PCR. Overall, we supposed that the prognostic model in this study was acceptable. Furthermore, we are planning to collect clinical samples for further verification.

In conclusion, the proposed irlncRNA pair-based signature has promising value in the prognostic prediction of LUAD. Furthermore, this prognostic model has great potential in the evaluation of tumor immune microenvironment and guiding individualized treatment regimens. Prospective evidence to further assess its accuracy and applicability are necessary in the future.

## Data Availability Statement

Publicly available datasets were analyzed in this study. This data can be found here: TCGA database, ImmPort database.

## Author Contributions

YJ designed the study. ZY download data from the TCGA and ImmPort database and did all the data analysis. MZ and TL drew all the figures. The tables were produced by JF and JD. The manuscript was drafted by ZY, MZ, TL, and JX. YJ supervised the overall workflow and critically revised the manuscript. YJ is the guarantor of this paper, taking responsibility for the integrity of the work as a whole, from inception to published article. All authors contributed to the article and approved the submitted version.

## Funding

This work was supported by the National Major Science and Technology Projects of China (CN):2019ZX09301001, and the Ministry of Science and Technology of the People’s Republic of China (CN):2020YFC0844300. The research sponsors did not participate in the study design, data collection, analysis and interpretation, and they were not involved in the writing of the manuscript and the decision to submit the manuscript for publication.

## Conflict of Interest

The authors declare that the research was conducted in the absence of any commercial or financial relationships that could be construed as a potential conflict of interest.

## Publisher’s Note

All claims expressed in this article are solely those of the authors and do not necessarily represent those of their affiliated organizations, or those of the publisher, the editors and the reviewers. Any product that may be evaluated in this article, or claim that may be made by its manufacturer, is not guaranteed or endorsed by the publisher.
