# Supplementary material for: Immune-Related lncRNA Pairs as Prognostic Signature and Immune-Landscape Predictor in Lung Adenocarcinoma
Source: Front Oncol. 2022 Jan 10;11:673567. doi: 10.3389/fonc.2021.673567 (PMC8784752; doi:10.3389/fonc.2021.673567)
Supplement: Supplementary file 3 [file Table_1.docx]

**Immune-Related lncRNA Pairs as** **Prognostic Signature and** **Immune-Landscape Predictor in Lung Adenocarcinoma**

Zhengrong Yin^1†^, Mei Zhou^1^^†^, Tingting Liao^1†^, Juanjuan Xu^1†^, Jinshuo Fan^1^, Jingjing Deng^1^, Yang Jin^1*^

**Supplementary materials**

**Table S1. 1209 immune-related lncRNAs identified by co-expression analysis** **(submitted as a separate excel file).**

**Table S2. 160 differentially expressed immune-related lncRNAs between tumor and (submitted as a separate excel file).**

**Table S3. The p value of comparing tumour infiltrating immune cells and risk sore (related to Figure 5)**

| **Symbol** | **p value** | **Symbol** | **p value** |
| --- | --- | --- | --- |
| B cell_TIMER | 0.0000 | T cell CD4+ effector memory_XCELL | 0.0007 |
| T cell CD8+_TIMER | 0.0220 | T cell CD8+_XCELL | 0.0007 |
| B cell memory_CIBERSORT | 0.0058 | T cell CD8+ central memory_XCELL | 0.0190 |
| T cell CD4+ memory resting_CIBERSORT | 0.0180 | Class-switched memory B cell_XCELL | 0.0010 |
| Monocyte_CIBERSORT | 0.0480 | Common lymphoid progenitor_XCELL | 0.0310 |
| Macrophage M0_CIBERSORT | 0.0001 | Common myeloid progenitor_XCELL | 0.0007 |
| Macrophage M2_CIBERSORT | 0.0260 | Myeloid dendritic cell activated_XCELL | 0.0024 |
| Mast cell activated_CIBERSORT | 0.0074 | Myeloid dendritic cell_XCELL | 0.0000 |
| B cell memory_CIBERSORT-ABS | 0.0013 | Cancer associated fibroblast_XCELL | 0.0027 |
| T cell CD4+ memory resting_CIBERSORT-ABS | 0.0007 | Granulocyte-monocyte progenitor_XCELL | 0.0002 |
| Monocyte_CIBERSORT-ABS | 0.0150 | Hematopoietic stem cell_XCELL | 0.0000 |
| Macrophage M0_CIBERSORT-ABS | 0.0025 | Macrophage_XCELL | 0.0120 |
| Macrophage M2_CIBERSORT-ABS | 0.0013 | Macrophage M2_XCELL | 0.0000 |
| Mast cell activated_CIBERSORT-ABS | 0.0015 | Mast cell_XCELL | 0.0000 |
| Macrophage M2_QUANTISEQ | 0.0000 | T cell NK_XCELL | 0.0051 |
| T cell regulatory (Tregs)_QUANTISEQ | 0.0002 | T cell CD4+ Th1_XCELL | 0.0190 |
| uncharacterized cell_QUANTISEQ | 0.0140 | T cell CD4+ Th2_XCELL | 0.0000 |
| T cell_MCPCOUNTER | 0.0230 | immune score_XCELL | 0.0000 |
| B cell_MCPCOUNTER | 0.0093 | stroma score_XCELL | 0.0033 |
| Myeloid dendritic cell_MCPCOUNTER | 0.0003 | microenvironment score_XCELL | 0.0000 |
| Neutrophil_MCPCOUNTER | 0.0085 | B cell_EPIC | 0.0110 |
| Endothelial cell_MCPCOUNTER | 0.0130 | Cancer associated fibroblast_EPIC | 0.0000 |
| Cancer associated fibroblast_MCPCOUNTER | 0.0007 | T cell CD8+_EPIC | 0.0000 |
| B cell_XCELL | 0.0130 | Macrophage_EPIC | 0.0170 |
| T cell CD4+ central memory_XCELL | 0.0050 |  |  |

**Table S4. The detail comparison results of correlation ship between tumour infiltrating immune cells and risk sore (related to Figure 6A).**

| **Symbol** | **Type** | **Correlation** | **p value** |
| --- | --- | --- | --- |
| B cell_TIMER | TIMER | -0.2722 | 0.0000 |
| T cell CD8+_TIMER | TIMER | -0.1258 | 0.0071 |
| Macrophage_TIMER | TIMER | -0.0957 | 0.0411 |
| Myeloid dendritic cell_TIMER | TIMER | -0.0942 | 0.0443 |
| B cell memory_CIBERSORT | CIBERSORT | -0.1600 | 0.0006 |
| T cell CD4+ memory resting_CIBERSORT | CIBERSORT | -0.1352 | 0.0038 |
| T cell CD4+ memory activated_CIBERSORT | CIBERSORT | 0.1168 | 0.0126 |
| Monocyte_CIBERSORT | CIBERSORT | -0.1188 | 0.0111 |
| Macrophage M0_CIBERSORT | CIBERSORT | 0.2113 | 0.0000 |
| Macrophage M2_CIBERSORT | CIBERSORT | -0.0999 | 0.0329 |
| Myeloid dendritic cell resting_CIBERSORT | CIBERSORT | -0.1481 | 0.0015 |
| Mast cell activated_CIBERSORT | CIBERSORT | -0.2241 | 0.0000 |
| Mast cell resting_CIBERSORT | CIBERSORT | 0.1425 | 0.0023 |
| B cell memory_CIBERSORT-ABS | CIBERSORT-ABS | -0.1843 | 0.0001 |
| T cell CD4+ memory resting_CIBERSORT-ABS | CIBERSORT-ABS | -0.1978 | 0.0000 |
| T cell CD4+ memory activated_CIBERSORT-ABS | CIBERSORT-ABS | 0.1108 | 0.0179 |
| Monocyte_CIBERSORT-ABS | CIBERSORT-ABS | -0.1522 | 0.0011 |
| Macrophage M0_CIBERSORT-ABS | CIBERSORT-ABS | 0.1527 | 0.0011 |
| Macrophage M2_CIBERSORT-ABS | CIBERSORT-ABS | -0.1779 | 0.0001 |
| Myeloid dendritic cell resting_CIBERSORT-ABS | CIBERSORT-ABS | -0.1671 | 0.0003 |
| Mast cell activated_CIBERSORT-ABS | CIBERSORT-ABS | -0.2429 | 0.0000 |
| Mast cell resting_CIBERSORT-ABS | CIBERSORT-ABS | 0.1250 | 0.0075 |
| B cell_QUANTISEQ | QUANTISEQ | -0.1048 | 0.0252 |
| Macrophage M2_QUANTISEQ | QUANTISEQ | -0.3364 | 0.0000 |
| NK cell_QUANTISEQ | QUANTISEQ | -0.1366 | 0.0035 |
| T cell CD4+ (non-regulatory)_QUANTISEQ | QUANTISEQ | 0.1072 | 0.0220 |
| T cell regulatory (Tregs)_QUANTISEQ | QUANTISEQ | -0.2379 | 0.0000 |
| uncharacterized cell_QUANTISEQ | QUANTISEQ | 0.1559 | 0.0008 |
| T cell_MCPCOUNTER | MCPCOUNTER | -0.1455 | 0.0018 |
| B cell_MCPCOUNTER | MCPCOUNTER | -0.1735 | 0.0002 |
| Monocyte_MCPCOUNTER | MCPCOUNTER | 0.0958 | 0.0409 |
| Macrophage/Monocyte_MCPCOUNTER | MCPCOUNTER | 0.0958 | 0.0409 |
| Myeloid dendritic cell_MCPCOUNTER | MCPCOUNTER | -0.2427 | 0.0000 |
| Neutrophil_MCPCOUNTER | MCPCOUNTER | -0.1386 | 0.0030 |
| Endothelial cell_MCPCOUNTER | MCPCOUNTER | -0.1738 | 0.0002 |
| Cancer associated fibroblast_MCPCOUNTER | MCPCOUNTER | 0.1674 | 0.0003 |
| Myeloid dendritic cell activated_XCELL | XCELL | -0.1840 | 0.0001 |
| B cell_XCELL | XCELL | -0.1375 | 0.0033 |
| T cell CD4+ naive_XCELL | XCELL | -0.0933 | 0.0464 |
| T cell CD4+ central memory_XCELL | XCELL | -0.1937 | 0.0000 |
| T cell CD4+ effector memory_XCELL | XCELL | -0.1967 | 0.0000 |
| T cell CD8+_XCELL | XCELL | -0.1785 | 0.0001 |
| T cell CD8+ central memory_XCELL | XCELL | -0.1263 | 0.0069 |
| Class-switched memory B cell_XCELL | XCELL | -0.1850 | 0.0001 |
| Common lymphoid progenitor_XCELL | XCELL | 0.1449 | 0.0019 |
| Common myeloid progenitor_XCELL | XCELL | -0.1913 | 0.0000 |
| Myeloid dendritic cell_XCELL | XCELL | -0.2495 | 0.0000 |
| Endothelial cell_XCELL | XCELL | -0.0982 | 0.0360 |
| Eosinophil_XCELL | XCELL | -0.1013 | 0.0305 |
| Cancer associated fibroblast_XCELL | XCELL | -0.1942 | 0.0000 |
| Granulocyte-monocyte progenitor_XCELL | XCELL | -0.2211 | 0.0000 |
| Hematopoietic stem cell_XCELL | XCELL | -0.3028 | 0.0000 |
| Macrophage_XCELL | XCELL | -0.1465 | 0.0017 |
| Macrophage M2_XCELL | XCELL | -0.3005 | 0.0000 |
| Mast cell_XCELL | XCELL | -0.2744 | 0.0000 |
| Monocyte_XCELL | XCELL | -0.1155 | 0.0136 |
| T cell NK_XCELL | XCELL | -0.1905 | 0.0000 |
| T cell CD4+ Th1_XCELL | XCELL | 0.1325 | 0.0046 |
| T cell CD4+ Th2_XCELL | XCELL | 0.2833 | 0.0000 |
| immune score_XCELL | XCELL | -0.2352 | 0.0000 |
| stroma score_XCELL | XCELL | -0.1857 | 0.0001 |
| microenvironment score_XCELL | XCELL | -0.2590 | 0.0000 |
| B cell_EPIC | EPIC | -0.1678 | 0.0003 |
| Cancer associated fibroblast_EPIC | EPIC | 0.2085 | 0.0000 |
| T cell CD8+_EPIC | EPIC | -0.2376 | 0.0000 |
| Endothelial cell_EPIC | EPIC | -0.1145 | 0.0144 |
| Macrophage_EPIC | EPIC | -0.1330 | 0.0044 |
